# Supplementary figures and images for: NS1-mediated DNMT1 degradation regulates human bocavirus 1 replication and RNA processing
Source: PLoS Pathog. 2024 Nov 14;20(11):e1012682. doi: 10.1371/journal.ppat.1012682 (PMC11594422; doi:10.1371/journal.ppat.1012682)

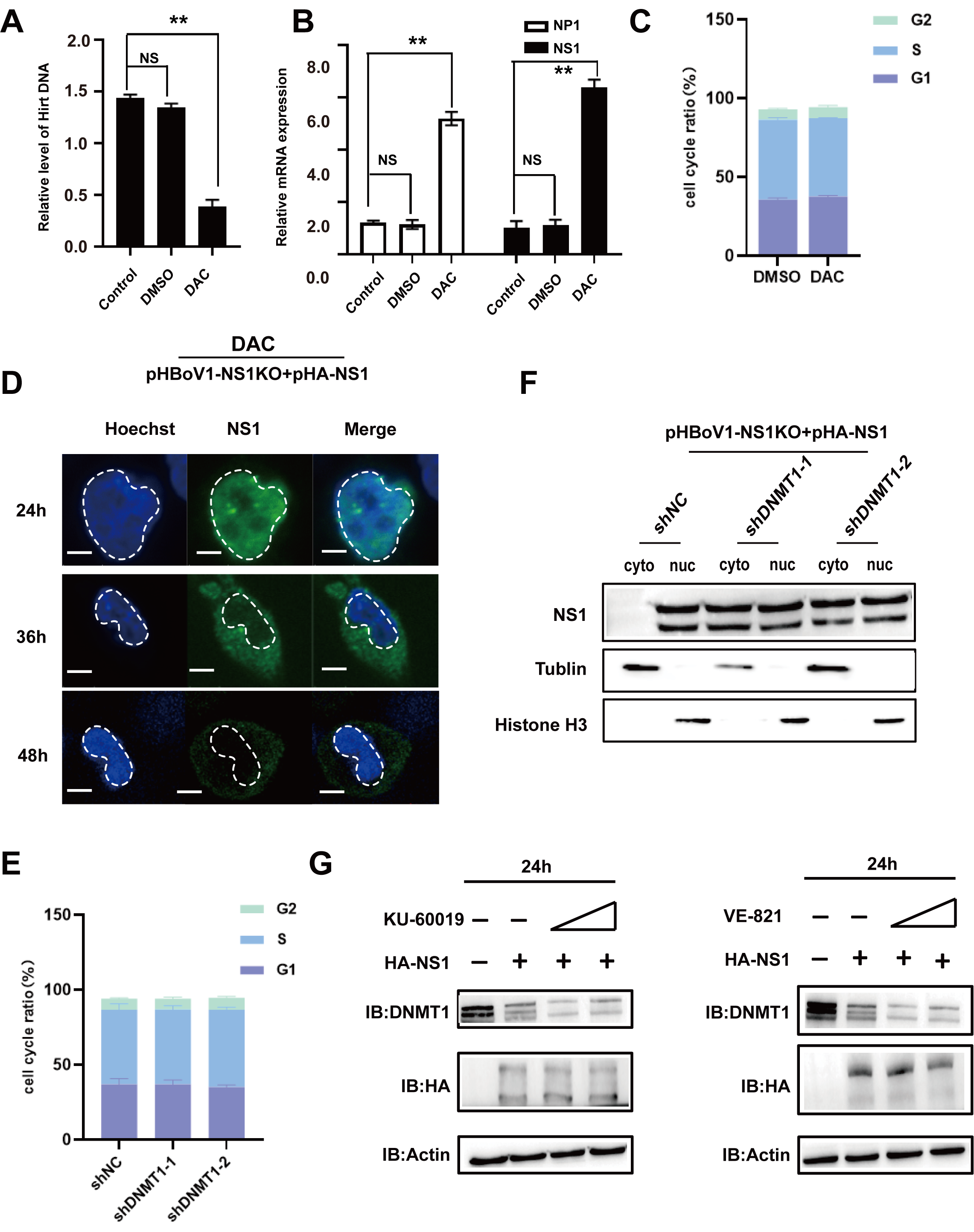

Supplement: S1 Fig — (A-B) The Dpn I digested Hirt DNAs (A) and the mRNAs expressing NS1 or NP1 (B) were quantified and analyzed through qRT-PCR in Calu-3 cells after DAC treatment. GAPDH was used as a control. *, p < 0.05, **, p < 0.01. (C) HEK293T cells were transfected with pHBoV1-WH and then treated with DMSO or 20uM DAC at 48h post-transfection, and the cell cycle of the treated cells were then analyzed by propidium iodide (PI) flow cytometry assay. (D) HEK293T cells were transfected with pHBoV-NS1KO and HA-NS1 and treated with DAC. To visualize the NS1, HA-NS1 was detected by IF using specific antibody against HA (green). The nuclei were stained with Hoechst 33258 (blue). The nuclear border is labeled by a white dashed line. (E) HEK293T cells with Stably knockdown of DNMT were selected as described in Materials and Methods. The cells were then transfected with pHBoV1-WH, and the cell cycle was analyzed by propidium iodide flow cytometry assay. (F) Subcellular fractionation of DNMT1 knockdown cells transfected with pHBoV-NS1KO and HA-NS1 was performed to validate the localization of HA-NS1 by western blot. The subcellular fractions of β-tublin and histone H3 indicated the efficient subcellular fractionation. (G) HEK293T cells were transfected with HA-NS1 and incubated with 20uM or 30uM KU-60019 or VE-821 (APE×BIO) for 24 h. The expression of DNMT1 and NS1 were measured by western blotting. (TIF) [file ppat.1012682.s001.tif]
