# Supplementary material for: NS1-mediated DNMT1 degradation regulates human bocavirus 1 replication and RNA processing
Source: PLoS Pathog. 2024 Nov 14;20(11):e1012682. doi: 10.1371/journal.ppat.1012682 (PMC11594422; doi:10.1371/journal.ppat.1012682)
Supplement: S1 Table — (DOCX) [file ppat.1012682.s002.docx]

| HBoV referene | Position | Strand | Pattern | Sequence | 5mC rate |
| --- | --- | --- | --- | --- | --- |
| JQ923422 | 207 | + | CHG | CAG | 0.81323 |
| JQ923422 | 1157 | + | CHG | CAG | 0.820717 |
| JQ923422 | 1382 | + | CHG | CAG | 0.772727 |
| JQ923422 | 1765 | + | CHG | CAG | 0.763705 |
| JQ923422 | 2819 | + | CHG | CAG | 0.782486 |
| JQ923422 | 3192 | + | CHG | CTG | 0.758621 |
| JQ923422 | 3525 | + | CHG | CAG | 0.758904 |
| JQ923422 | 4090 | + | CHG | CTG | 0.721622 |
| JQ923422 | 4314 | + | CHG | CTG | 0.655773 |
| JQ923422 | 4440 | + | CHG | CTG | 0.667674 |
| JQ923422 | 4872 | + | CHG | CAG | 0.665625 |
| JQ923422 | 209 | - | CHG | CTG | 0.713355 |
| JQ923422 | 1159 | - | CHG | CTG | 0.75853 |
| JQ923422 | 1384 | - | CHG | CTG | 0.782946 |
| JQ923422 | 1767 | - | CHG | CTG | 0.728814 |
| JQ923422 | 2821 | - | CHG | CTG | 0.761438 |
| JQ923422 | 3194 | - | CHG | CAG | 0.72 |
| JQ923422 | 3527 | - | CHG | CTG | 0.839216 |
| JQ923422 | 4092 | - | CHG | CAG | 0.743802 |
| JQ923422 | 4316 | - | CHG | CAG | 0.677419 |
| JQ923422 | 4442 | - | CHG | CAG | 0.672365 |
| JQ923422 | 4874 | - | CHG | CTG | 0.667802 |

**S1 Talbe** HBoV methylation sites after DpnI digestion
