# Supplementary material for: NS1-mediated DNMT1 degradation regulates human bocavirus 1 replication and RNA processing
Source: PLoS Pathog. 2024 Nov 14;20(11):e1012682. doi: 10.1371/journal.ppat.1012682 (PMC11594422; doi:10.1371/journal.ppat.1012682)
Supplement: Attachment — Submitted filename: DS-Point by point responses-final.docx [file ppat.1012682.s003.docx]

**Point-by-point responses to the reviewers’ comments (PPATHOGENS-D-24-00020)**

**Reviewers’ Comments:**

Reviewer #1:

In this manuscript, Qin et al. have attempted to elucidate the mechanism by which DNA methylation regulates the life cycle of the pediatric respiratory parvovirus human bocavirus (HBoV). Since the mechanisms regulating efficient parvovirus chromatinization and subsequent gene expression remain unknown, this study attempts to connect the viral life cycle to the methyltransferase DNMT1. While the broader findings of the manuscript regarding parvovirus life cycle are interesting, a major weakness of the study is its reliance on transfected HBoV infectious clone into 293T cells. This is particularly concerning since the transfected infectious clone genome itself is methylated by bacteria, and replication generates multiple genomic forms in the nuclear compartment. This makes it difficult to interpret whether the methylation occurs on the viral genome or the infectious clone. Additionally, the authors need to address the generalizability of their discoveries during viral infection of appropriate models of HBoV infection. Specific major and minor comments regarding the manuscript have been described below.

Response:

In this study, we utilized the differentiated airway epithelium cultured at an air-liquid interface as the HBoV1 infection model [1]. Human bronchial epithelial Calu-3 cells were cultured in semipermeable membrane inserts (0.6 cm²; Millicell-PCF; Millipore), with approximately 10⁴ cells per insert. However, the protein or RNA yields from these inserts are insufficient for Southern blotting, bisulfite sequencing, Northern blotting, or RNA protection assays (RPA), which typically require about 10⁷ cells per experiment. Consequently, scaling up the air-liquid interface cell culture would require increasing the culture volume by 100 to 1000 times, demanding substantial resources and additional funding that are currently beyond our reach. Therefore, we leveraged the well-characterized HBoV transfection system using HEK293T cells for this study.

Although HEK293T cells are not fully permissive to HBoV infection due to the absence of the virus receptor, transfection of HBoV DNA into these cells mimics replication in natural host cells. This system efficiently generates replication products such as double replicative form DNA, monomer replicative form DNA, and single-stranded DNA. Transfection also yields high titers of progeny virus particles, which can infect polarized primary human airway epithelia (natural host cells). This indicates that HEK293T cells support most aspects of the HBoV life cycle, except for viral entry. Unfortunately, the virus receptor remains unidentified. We utilized this transfection system to study HBoV DNA replication and RNA processing, as it allows us to collect sufficient viral DNA and RNA for Southern blotting, bisulfite sequencing, Northern blotting, and RPA.

To address concerns about genome methylation potentially originating from bacteria and to ensure findings are specific to HEK293T cells, we conducted additional experiments as suggested by reviewers. We also explicitly state the limitations of this work in the revised manuscript, immediately following the discussion section, to ensure that all unresolved issues are clearly communicated to readers.

Major Comments

1. Bisulfite sequencing of 293T cells transfected with the HBoV infectious clone is likely to yield heterogeneous results, some of which are methylation marks on the plasmid and some are on the replicating viral genome. Line 102 states that this was done in “cells transfected with the HBoV infectious clone”, whereas Line 614 states that these studies were carried out “48 h post-infection”. Which is it? If this assay was performed in the transfected cells, then they need to be carried out during HBoV infection. The assay should also be carried out in an independent cell line model to demonstrate the generalizability of the methylation mark.

Response: Thank you for your professional suggestions. To address the issue of heterogeneous methylation results from bacterial contamination and HEK293T cells, we revised our protocol and repeated the whole genome bisulfite sequencing. First, to minimize plasmid methylation, we used JM110 Chemically Competent Cells (Beijing Tsingke, TSC-C08), which are Dam and Dcm methylase-deficient E. coli strains, for plasmid amplification. Second, we prepared Hirt DNA and removed plasmid DNA through DpnI digestion prior to whole genome bisulfite sequencing. Despite the plasmids being amplified from JM110 cells, they remained sensitive to DpnI digestion. As shown in the revised data (Fig. 1A), the majority of methylation sites detected post-DpnI digestion were consistent with those identified before the revision. However, we observed a reduction in the number of methylation sites in the DpnI digestion group. The figure and results have been updated accordingly. Thank you for your attention to this matter.

Line 675, “48h post-infection” were changed to “48h post transfection” in the revised manuscript.

2. Since DAC impacts cell cycle progression, the authors need to show what is the impact of DAC treatment on cell cycle entry into G1 and S phases during HBoV transfection/infection. The modest increase in NP1 levels in Fig. 1B might just be a result of the secondary impacts of DAC treatment on host cell cycle progression.

Response: Thank you for your suggestions. As shown in the data below, neither the G1 nor S phases of HEK293T cells were affected by DAC treatment in the presence of HBoV transfection after 48 hours. Therefore, the increased viral protein expression observed with DAC treatment is not due to any effects of DAC on the cell cycle.

Fig.S1C HEK293T cells were transfected with pHBoV1-WH and then treated with either DMSO or 20 µM DAC 48 hours post-transfection. The cell cycle of the treated cells was subsequently analyzed using propidium iodide (PI) flow cytometry.

3. The partial GAPDH bands in Fig. 1C make it difficult to interpret the loading on this western blot. It is also inappropriate to quantify the signal intensity of a partial band in lane 4 of Fig. 1C as well as take ratio to that of DNMT1. I have a similar concern about the partial banding in the DNMT3A levels in lane 4 of Fig. 1C.

Response: Thank you for your suggestions. We conducted additional independent experiments to assess whether DNMT1 and other DNMT proteins were downregulated 48 hours post-transfection. Our results indicate that DAC treatment significantly reduced DNMT1 expression compared to the control, while the levels of other DNMT proteins remained relatively unchanged. The original panel has been replaced with the updated results, as shown below.

4. It is unclear why the NS1 levels are decreasing as transfection progresses from 24 hpi to 48 hpi (compare Fig. 1C, lane 2 to lane 4). This observation makes the conclusion from Fig. 1C impossible to interpret.

Response: In other independent experiments, NS1 levels did not decrease, as shown above. However, DNMT1 expression significantly decreased 48 hours after transfection with HBoV DNA. The detection of NS1 indicates the successful HBoV transfection.

5. In Fig. 1D, the authors need to show how NS1/NP1 levels are changing during infection of Calu-3 cells. It is also critical to provide the levels of a DNMT that does not change as a control. Any of the DNMT’s that are unchanged in Fig. 1C would suffice, but they should demonstrate specificity of the impact of HBoV infection that is specific to DNMT1.

Response: Thank you for your suggestions. We assessed the expression of NS1, NP1, and DNMT3A at 72 and 120 hours post-infection in Calu-3 cells. NS1 and NP1 were efficiently expressed. In contrast, DNMT1 expression was dramatically reduced following infection, while DNMT3A levels remained relatively unchanged. The original panel has been replaced with the updated results, as shown below.

6. How does DAC treatment impact HBoV NS1 and NP1 during infection of Calu-3 cells?

Response: Thanks for your suggestions. We performed qRT-PCR analysis to examine the mRNA expression levels of NS1 and NP1 in Calu-3 cells. Consistent with observations in HEK293T cells (Fig. 2C and Fig. 3B), DAC treatment also led to an increase in the mRNAs of NS1 and NP1 in Calu-3 cells (Fig. S1B).

7. While the findings of Fig. 2A measuring HBoV genome replication are interesting, this does not address the possibility of methylation marks on the plasmid infectious clone showing an aberrant phenotype. It is therefore crucial for the authors to perform a Southern blot during HBoV infection of Calu-3 cells in the presence of DAC to confirm that replication of the virus during infection is regulated by genome methylation.

Response: Thanks for your suggestions. Southern blotting requires several million cells per sample, but the DNA collected from the infection model (inserts) is insufficient for this analysis. Instead, we performed qPCR analysis on the Hirt DNA extracted from Calu-3 cells (Fig. S1A). Our results show that DAC treatment reduced HBoV genome replication, as illustrated below.

8. It is impossible to interpret the findings of Fig. 2D, 2E. The authors need to show the NS1 and NP1 localization to nuclear sites at earlier stages of expression prior to complete nuclear takeover. Importantly, these experiments must be performed during HBoV infection in Calu-3 cells. The nuclear border needs to be demarcated by a white dashed line to indicate which phenomenon are nuclear and which are cytoplasmic.

Response: Thank you for your suggestions. To visualize the localization of NS1 and NP1 at earlier stages, we examined their nuclear localization using immunofluorescence staining at 24, 36, and 48 hours post-transfection in HEK293T cells treated with DAC. NS1 was predominantly localized in the nucleus up to 24 hours post-transfection, but by 36 hours, most of NS1 had relocated to the cytoplasm. Unfortunately, Calu-3 cells are not suitable for our current immunostaining protocol due to their non-monolayer nature.

HEK293T cells were transfected with pHBoV-NS1KO and HA-NS1 and treated with DAC. To visualize the NS1, HA-NS1 was detected by IF using specific antibody against HA (green). The nuclei were stained with Hoechst 33258 (blue). The nuclear border is labeled by a white dashed line.

9. Studies on RNA splicing and polyadenylation have been performed exclusively in 293T cells transfected with the plasmid form of HBoV. Are these findings phenocopied in other cell lines and during HBoV infection?

Response: Thank you for your suggestions. HEK293T cells are commonly used for transfection of HBoV DNA and virus production [1]. We also transfected HBoV DNA into two other cell lines, HeLa and Huh7, and quantified DNA synthesis by qPCR (A). Compared to HEK293T cells, DNA synthesis in HeLa and Huh7 cells was notably lower (A), suggesting a deficiency in DNA replication in these cell lines. We further analyzed the effects of DAC treatment on RNA splicing at the D1 and D3 sites by RT-PCR in Huh7 cells. DAC treatment enhanced RNA expression and splicing at these sites, as shown below (B and C).

Additionally, RT-PCR analysis demonstrated that DAC treatment enhanced RNA splicing at the D1 site in Calu-3 cells, as shown below.

10. The authors need to show whether DAC treatment and methylation levels of the host cells impact the recruitment of the transcription machinery to the viral genome. This needs to be done with chromatin immunoprecipitation of RNA polymerase II on the viral genome.

Response: We performed ChIP analysis of NS1 in HBoV-transfected cells following DAC treatment as we did before [2]. The results showed a significant reduction in NS1 recruitment to the HBoV genome under DAC treatment, while recruitment of POL II was dramatically increased (Fig. 6E). This change was associated with decreased viral DNA replication and increased RNA expression. The updated data has been added to Fig. 6 in the revised manuscript.

Fig.6 A and E HEK293T cells were transfected with the indicated plasmids and treated with either DAC (5-Aza-2'-deoxycytidine) or DMSO (dimethyl sulfoxide). Chromatin immunoprecipitation (CHIP) was performed 48 hours post-transfection using either an HA antibody or a POL II antibody as we did before [2]. The amount of NS1-associated HBoV DNA was quantified by qPCR, employing four pairs of primers that span the viral genome, as previously described.

11. What is the impact of DNMT knockdown on cell cycle entry? This might explain the attenuated impact on HBoV replication. The authors need to show these control assays. Additionally, the knockdown studies must be performed during HBoV infection.

Response: As shown below, the G1 and S phases of the cell cycle were not affected by DNMT1 knockdown. The reduced replication observed with either DAC treatment or DNMT1 knockdown is therefore not due to changes in the cell cycle phases.

HEK293T cells with stable DNMT knockdown were selected according to the procedures outlined in the Materials and Methods section. Parallel to this, shNC control cells were prepared. Following transfection with pHBoV1-WH, the cell cycle was analyzed using propidium iodide flow cytometry.

Minor Comments

1. In addition to the locations described on the table and the line diagram in Fig. 1A, the results of the bisulfite sequencing should also represent the +/- polarity. These findings are a critical resource for the HBoV field and should be scaled as closely as possible to actual distances along the HBoV genome.

Response: Fig.1A has been revised accordingly as suggested.

2. The bands on Fig. 3B are overexposed making it difficult to interpret the findings of this assay. The authors should provide images of short exposure and long-exposure, especially if they are going to quantify the bands.

Response: The figures has been replaced by short exposure image of independent experiment.

3. Line 159 – the phrase “major player” is a little informal.

Response: Line 168, it has been changed to major methyltransferase.

4. Line 166 – “Notebly” should be “Notably”

Response: Line 177, it has been corrected.

5. Would DAC treatment during shRNA-mediated knockdown of DNMT1 have an additive effect on viral life cycle? The authors should address this possibility in the text.

Response: It has been discussed in the revised manuscript. Line 246-247, However, whether DAC treatment on the DNMT1 knockdown cells have a synergistic effect on virus replication is unknown.

6. In Fig. 4D and 4E, the nuclear border needs to be demarcated by a dashed line to indicate more clearly what is cytoplasmic and what is nuclear. These findings should additionally be presented by independent biochemical fractionation such as those shown in Fig. 2F and 2G.

Response: The nuclear border has been indicated in the revised manuscript as below (Fig.4D and E).

The cytoplasmic localization of NS1 in HBoV transfection were further confirmed by subcellular fractionation experiments (Fig.S1F).

Fig.S1F Stably knockdown of DNMT1 was performed as before. Subcellular fractionation of DNMT1 knockdown cells transfected with pHBoV-NS1KO and HA-NS1 was performed to validate the localization of HA-NS1 by western blot. The subcellular fractions of β-tublin and histone H3 indicated the efficient subcellular fractionation.

7. The overall schematic of the findings in Fig. 6 are very difficult to follow. It tries to summarize all the detailed findings of the paper rather than illustrate how 5mC regulates HBoV life cycle.

Response: The model has been revised as suggested.

Reviewer #2: Strengths/Novelty/Significance:

•Qin et al. utilized the HBoV model to characterize the role of DNA methylation in parvovirus DNA replication and RNA processing.

•The study effectively characterized and showed that DNA methylation inhibition with 5-aza-2′-deoxycytidine (DAC) in HEK293T cells and Calu-3 cells coupled DNMT1 shRNA knockdown sufficiently revealed alteration in parvoviral DNA replication and RNA processing.

•Similarly, DNA methylation inhibition with 5-aza-2′-deoxycytidine (DAC) in HEK293T cells and Calu-3 cells coupled DNMT1 shRNA knockdown indicated altered expression of NS1 and NP1 protein in addition to predominant NS1 cytoplasmic localization.

•Immunoprecipitation of DNMT1 with NS1 added credence to the potential role of DNA methyltransferase in parvoviral gene expression and host-cell interaction.

•The results provided evidence that NS1 expressed from the infectious clone pHBoV1-WH, and epitope-tag construct (HA-NS1 and FLAG-NS1) modulated the DNMT1 depletion with restoration to basal levels after MG132 treatment in a proteasome-dependent manner.

•The study explores novel questions that have not been comprehensively explored in parvoviral biology and host-cell interaction, with potential implications for the advancement of parvoviral gene therapy.

•This study will expand the frontiers of parvovirus and DNA viruses host cell interaction.

Weakness:

Overall, while the study showed a well-characterized phenotype with well-designed experiments with DNA methylation chemical inhibition, DNMT1 shRNA knockdown, RNase protection assays, Southern blot, immunoprecipitation, and immunofluorescence assays, there are few experiments with emphasis to efficiently decipher and dissect the mechanisms associated with the characterized phenotypes.

I’m of the opinion that additional experiments suggested below to explain the mechanisms associated with the described and characterized results will significantly add value to the characterized results for future submission to the journal.

1. Experiment to identify which specific HBoV NS1 isoforms and NS1 functional domains (DNA binding domain, helicase domain, or intrinsically disordered region) interact with DNMT1 to mediate the observed depletion and degradation.

Response: HBoV generates several NS isoforms through alternative splicing. To determine which specific isoforms interact with DNMT1 and mediate its degradation, cDNAs of four NS isoforms were cloned and expressed in HEK293T cells. NS1 is translated from the D2-A2 spliced RNA. NS2 is derived from the D1'-A1 spliced RNA and lacks the helicase domain. NS3 is translated from the D1-A1' spliced RNA and is missing the DNA binding domain. NS1-70 is produced from the D2-A2 retained RNA, which terminates at a stop codon in the intron region, resulting in a deletion of the transactivation domain (Fig. 6A).

Since the NS1 cDNA included the intact D1'-A1 intron, it was spliced to produce the NS2 protein (Fig. 6B). Although the amount of NS3 RNA was comparable to that of other isoforms, NS3 protein expression was significantly lower (Fig. 6B). NS1, NS2, and NS1-70 all efficiently mediated DNMT1 degradation (Fig. 6C), and both NS1 and NS2 directly interacted with DNMT1 (Fig. 6D). Thus, the transactivation domain is necessary for interaction with DNMT1 but is not required for NS1-mediated DNMT1 degradation.

Thank you for your attention to this matter.

Figure 6. Both NS1 and NS2 interacted with DNMT1 and promote DNMT1 degradation. (A) the NS isoforms are diagramed under the HBoV genome. (B and C) HEK293T cells were transfected with the indicated plasmids. The expressions of HA-NS1, HA-NS2, HA-NS3 or HA-NS1-70 were examined by western blotting as described above (B). HA-NS1, HA-NS2 or HA-NS1-70 promoted DNMT1 degradation (C). (D) co-IP was performed with an anti-DNMT1 antibody at 48 h post-transfection to evaluate the interactions with HA-NS1, HA-NS2 or HA-NS1-70.

2.Recently, Haubold et al., reported that ectopically expressed MVM NS1 and NS2 proteins are sufficient to cause host-cell replication stress. However, there are no experiments in the study to explore the potential role of DNA damage response in NS1-mediated DNMT1 depletion.

DNMT1 expression pattern and kinetics in HBoV infection with DDR kinase (ATM and ATR) inhibitors and UV-inactivated HBoV genomes may provide additional insights about the mechanism utilized for DNMT1 depletion.

Response: NS1-mediated degradation of DNMT1 was slightly enhanced by both the ATR inhibitor KU-60019 and the ATM inhibitor VE-821. ATR- or ATM-dependent DNA damage responses are also triggered by parvovirus infections and play a crucial role in viral DNA replication mediated by the NS1 protein. These findings suggest a cross-talk between DNMT1 and DNA damage responses; however, the degradation of DNMT1 by NS1 appears to be independent of the DNA damage responses induced by the virus.

HEK293T cells were transfected with HA-NS1 and incubated with 20uM or 30uM KU-60019 or VE-821 (APE×BIO) for 24 h. The expression of DNMT1 and NS1 were measured by western blotting.

3. Since NS1 generally activates parvovirus promoters and drives promoter transcriptional activity, time course and pulse-chase experiments to elucidate the mechanism associated with the predominant NS1 cytoplasmic localization during 5-aza-2′-deoxycytidine (DAC) in HEK293T cells will elucidate and provide insights on the discrepancy between elevated NS1 and NP1 transcripts while the predominant transcriptional activator is localized in the cytoplasm.

Response: Thank you for your suggestions. To visualize the localization of NS1 and NP1 at earlier stages, we examined their nuclear localization using immunofluorescence staining at 24, 36, and 48 hours post-transfection in HEK293T cells treated with DAC. NS1 was predominantly localized in the nucleus up to 24 hours post-transfection, but by 36 hours, most of NS1 had relocated to the cytoplasm. Unfortunately, Calu-3 cells are not suitable for our current immunostaining protocol due to their non-monolayer nature.

HEK293T cells were transfected with pHBoV-NS1KO and HA-NS1 and treated with DAC. To visualize the NS1, HA-NS1 was detected by IF using specific antibody against HA (green). The nuclei were stained with Hoechst 33258 (blue). The nuclear border is labeled by a white dashed line.

4. HBoV P5 promoter chromatin immunoprecipitation (CHIP) assay with pHBoV1-WH, pHBoV1-NS1KO and during 5-aza-2′-deoxycytidine (DAC) treatment on the promoter activity and NS1 occupancy during these conditions.

Response: We performed ChIP analysis of NS1 in HBoV-transfected cells following DAC treatment. The results showed a significant reduction in NS1 recruitment to the HBoV genome under DAC treatment, while recruitment of POL II was dramatically increased (Fig. 6E). This change was associated with decreased viral DNA replication and increased RNA expression. The updated data has been added to Fig. 6 in the revised manuscript.

Fig.6 A and E HEK293T cells were transfected with the indicated plasmids and treated with either DAC (5-Aza-2'-deoxycytidine) or DMSO (dimethyl sulfoxide). Chromatin immunoprecipitation (CHIP) was performed 48 hours post-transfection using either an HA antibody or a POL II antibody as we did before [2]. The amount of NS1-associated HBoV DNA was quantified by qPCR, employing four pairs of primers that span the viral genome, as previously described.

5. The discussion of the potential mechanism involved in the NS1-mediated DNMT1 degradation is sparse therefore, additional text to explain potential and likely mechanisms involved (E3 ligases involved) will comprehensively improve the manuscript.

Response: A short discussion was added in Lines 258-261. “NS1-mediated degradation of DNMT1 was slightly enhanced by both ATR inhibitor KU-60019 and ATM inhibitor VE-821 (Fig.S1G), indicating a possible cross talk with virus induced DNA damage responses but not fully required for DNMT1 degradation. As ubiquitin-proteasome pathway is the most potential mechanism involved in the NS1-mediated DNMT1 degradation, further study of the E3 ligase involved enzymatic cascade is of interest in the future.”

Below are comments that are unclear and typographical errors that should clarified and corrected for future submission.

Comments:

1. Line 116-119: Is the HBoV methylation pattern, including methylation sites in Calu-3 cells, similar to results observed in HEK293T cells?

Response: In this study, we utilized the differentiated airway epithelium cultured at an air-liquid interface as the HBoV1 infection model [1]. As detailed in the Materials and Methods section, human bronchial epithelial Calu-3 cells were cultured in semipermeable membrane inserts (0.6 cm²; Millicell-PCF; Millipore), with approximately 10^4^ cells seeded per insert. The amount of protein or RNA collected from these inserts was insufficient for bisulfite sequencing. To replicate the findings in an infection model, it would be necessary to scale up the air-liquid interface cell culture, which would require substantial effort and additional funding resources that are currently beyond our reach. However, we have clearly stated in the "Limitations" section of the revised manuscript that“the results and conclusions were mainly based on the transfection of HBoV infectious clone in HEK293T cells”to ensure this limitation is visible to all readers.

2. Line 133-134: While there is increased NS1 and NP1 mRNA expression, is it related to increased promoter transcription activity? P5 Chromatin immunoprecipitation assay may provide insights into the promoter activity

Response: We performed ChIP analysis of NS1 in HBoV-transfected cells following DAC treatment. The recruitment of NS1 to the HBoV genome was significantly reduced with DAC treatment, whereas POL II recruitment was dramatically increased (Fig. 6E). This resulted in decreased viral DNA replication and increased RNA expression. The updated data are now included in Figure 6 of the revised manuscript.

3. Line 141-144: The mechanism and potential reasons for NS1 cytoplasmic location after DAC treatment are unexplained in the results and discussion.

Response: The mechanism underlying the cytoplasmic retention of NS1 following DAC treatment is not fully understood. Exportin (CRM1) and importin α/β have been implicated in the shuttling of PPV NS1 between the nucleus and cytoplasm [3]. Therefore, it is possible that inhibition of DNA methylation by DAC treatment or DNMT1 knockdown may affect the functional pathways of these transport receptors, leading to the cytoplasmic localization of NS1.

4. Line 180-182: The statement “NS1 start codon-mutated virus failed to downregulate DNMT1” is unclear because the pHBoV-NS1KO construct will not express NS1. Therefore, there is no genome replication required for virion production.

Response: Line 192, a clause of “which did not express NS1 for viral DNA replication” was added.

Typographical errors

1. Line 136: Immunofluorescence should be changed to immunofluorescence

Response: Line 144, it has been corrected.

2. Line 137: This statement “the localization of NP1 was exclusively expressed in the nucleus” is unclear, the statement should be clarified

Response: Line 145, it has been changed to “NP1 exclusively localized in the nucleus in the presence or absence of DAC treatment”

3. Line 147: The phrase” closely correlated with alternative RNA splicing is unclear.

Response: Line 156, it has been changed to “regulates alternative RNA splicing”.

4. Line 166: “Notebly” should be “notably”

Response: Line177, it has been corrected.

**References**

1.	Yan Z, Deng X, Qiu J. Human Bocavirus 1 Infection of Well-Differentiated Human Airway Epithelium. Curr Protoc Microbiol. 2020;58: e107. doi:10.1002/cpmc.107

2.	Zhang X, Guo J, Xu H, Ding S, Liu L, Chen Z, et al. NS1-mediated enhancement of MVC transcription and replication promoted by KAT5/H4K12ac. J Virol. 2024;98: e0169523. doi:10.1128/jvi.01695-23

3.	Cao L, Fu F, Chen J, Shi H, Zhang X, Liu J, et al. Nucleocytoplasmic Shuttling of Porcine Parvovirus NS1 Protein Mediated by the CRM1 Nuclear Export Pathway and the Importin α/β Nuclear Import Pathway. J Virol. 2022;96: e0148121. doi:10.1128/JVI.01481-21
